# Management and patient safety of complex elderly patients in primary care during the COVID-19 pandemic in the UK—Qualitative assessment

**DOI:** 10.1371/journal.pone.0248387

**Published:** 2021-03-29

**Authors:** Ahmed Alboksmaty, Sonia Kumar, Ravi Parekh, Paul Aylin

**Affiliations:** 1 NIHR Patient Safety Translational Research Centre (PSTRC), Imperial College London, London, United Kingdom; 2 Undergraduate Primary Care Education and Medical Education Innovation and Research Centre (MEdIC), Imperial College London, London, United Kingdom; 3 Medical Education Innovation and Research Centre (MEdIC), Imperial College London, London, United Kingdom; 4 Epidemiology and Public Health, Director Dr Foster Unit, School of Public Health, Imperial College London, London, United Kingdom; Massachusetts General Hospital, UNITED STATES

## Abstract

**Objectives:**

The study aims to investigate GPs’ experiences of how UK COVID-19 policies have affected the management and safety of complex elderly patients, who suffer from multimorbidity, at the primary care level in North West London (NWL).

**Design:**

This is a service evaluation adopting a qualitative approach.

**Setting:**

Individual semi-structured interviews were conducted between 6 and 22 May 2020, 2 months after the introduction of the UK COVID-19 Action Plan, allowing GPs to adapt to the new changes and reflect on their impact.

**Participants:**

Fourteen GPs working in NWL were interviewed, until data saturation was reached.

**Outcome measures:**

The impact of COVID-19 policies on the management and safety of complex elderly patients in primary care from the GPs’ perspective.

**Results:**

Participants’ average experience was fourteen years working in primary care for the NHS. They stated that COVID-19 policies have affected primary care at three levels, patients’ behaviour, work conditions, and clinical practice. GPs reflected on the impact through five major themes; four of which have been adapted from the Safety Attitudes Questionnaire (SAQ) framework, changes in primary care (at the three levels mentioned above), involvement of GPs in policy making, communication and coordination (with patients and in between medical teams), stressors and worries; in addition to a fifth theme to conclude the GPs’ suggestions for improvement (either proposed mitigation strategies, or existing actions that showed relative success). A participant used an expression of “infodemic” to describe the GPs’ everyday pressure of receiving new policy updates with their subsequent changes in practice.

**Conclusion:**

The COVID-19 pandemic has affected all levels of the health system in the UK, particularly primary care. Based on the GPs’ perspective in NWL, changes to practice have offered opportunities to maintain safe healthcare as well as possible drawbacks that should be of concern.

## Introduction

Primary care is fundamental to a resilient healthcare system, ultimately providing safe and timely services in all settings, including outbreaks and emergencies [1]. Providing safe primary care is more challenging for elderly patients with multimorbidity, who will be referred to as complex elderly patients in this study. Previous literature has defined multimorbidity as the coexistence of two or more chronic health conditions in an individual [2]. Even though multimorbidity is not exclusive to a specific age group, evidence shows that it is higher amongst elderly people aged 65 years and above, who represent nearly 20% of the overall UK population [3–6]. Healthcare management for those patients requires a collaborative approach to provide closer monitoring and constitutes the majority of GPs’ workload in the UK [2, 7].

Recently, the COVID-19 pandemic has highlighted the challenges of frailty and safety of the elderly population as a global health concern [8]. The elderly population are not only at a higher risk of contracting COVID-19 and sufferring a worse outcome from it, they may also be at risk of neglect of their pre-exisiting chronic conditions or being lost to follow up [9]. To combat the pandemic and protect people, many countries have introduced strict public health measures, such as shielding of complex elderly patients [10, 11].

The UK National Health Service (NHS) adopted a set of policies for primary care as a response to the pandemic. These new policies include establishing a remote “total triage” model in general practice via phone or online platforms; preparing for an expected increase in home-visit consultations; and prioritising services and patient groups according to risk [11]. These policies are aligned with the introduction of shielding and social isolation measures across the country for at-risk groups [12].

While these new policies were designed to enhance the NHS capacity to suppress the pandemic, there has been growing concern regarding possible unintended consequences [12]. A notable reduction in the overall number of GP appointments has been reported since the beginning of the pandemic. Face-to-face appointments in primary care fell by 30%, while remote consultations recorded a substantial rise [13]. Hospitals reported 30% fewer emergency admissions compared to the same period in 2019 [14]. However, these figures should be interpreted with caution given the potential latent deterioration in health, particularly amongst patients with multiple chronic conditions [15].

As primary care is the entry point for the health system, it has been pivotal to the pandemic response [16, 17]. This study explores the perspective of GPs as frontline health workers in maintaining the safety of complex elderly patients in primary care during the COVID-19 pandemic in the UK.

## Study aim and objectives

This study aims to investigate GPs’ experiences of how the UK COVID-19 policies have affected the management and safety of non-COVID elderly patients with multimorbidity in primary care.

### Objectives

1. To acquire a realistic picture from a practitioner perspective of how COVID-19 has impacted primary care services
2. To investigate the challenges and gaps in the management and safety of complex elderly patients in primary care during COVID-19
3. To explore potential solutions for the identified gaps of how to ensure effective management and safety of complex elderly patients in primary care during COVID-19

## Methodology

### Study design

This is a service evaluation adopting a qualitative approach. Individual semi-structured interviews were conducted with GPs who deliver primary care in North West London (NWL) locality during the COVID-19 pandemic.

### Theoretical framework and interview guide

The COVID-19 policies have significantly changed the delivery of healthcare, especially at the primary care organisational level [11]. Two theoretical frameworks were the base for drafting a primary interview guide to explore these policies’ impact.

The first framework was the Fourth Generation Evaluation by Guba and Lincoln [18], which has been used earlier in similar contexts in primary care [19, 20]. It guides the evaluation of new policies’ effectiveness and impact based on the actual context at the time of implementation [21]. The second was adapted from the Safety Attitudes Questionnaire (SAQ) [22]; it is one of the most widely used and rigorously validated tools for assessing patient safety in different healthcare settings [23].

The primary interview guide was pilot-tested with three additional participants, an academic, a GP with a managerial role, and a primary care research expert. This was to ensure the validity and comprehensiveness of the interview guide to practically explore the study objectives. A final flexible interview guide (Table 1) was developed and reassessed by the research team. An added standpoint was included to explore improvement actions from the GPs’ perspective in overcoming identified gaps in service and offering practical solutions.

**Table 1:** Interview guide.

Interview guide
**1 How have primary care and your role changed since the COVID-19 outbreak?**
**1.1** What do you currently see as working well, and not so well?
**1.2** How do you think current primary care changes may affect your patients in primary care (positively or negatively)?
**2 What are the current issues in primary care with the management and safety of elderly patients with complex conditions with the new COVID-19 policies in place?**
**2.1** What are the implications of using a remote total triage system and limiting face-to-face consultations?
**2.2** How do you respond if you receive a patient request for “urgent medical attention” related to a pre-existing chronic condition?
**2.3** How has patient isolation/shielding impacted patients access or presentation to primary care?
**2.4** Are there any particular groups of complex elderly patients you are most concerned about?
**3 How has the coordination and communication between patients, health workers and managers in primary care changed during the COVID-19 pandemic?**
**3.1** Both at a practice, CCG and PCN level
**3.2** Could this have an impact on the care of elderly patients with complex conditions?
**4 What do you think about the involvement of GPs and frontline health workers in the UK policy making during the COVID-19 outbreak?**
**4.1** Do you think the safety of elderly patients with complex conditions has been adequately considered?
**5 How do you think (*suggestions*) you can ensure and maintain safety of elderly patients with existing complex conditions in primary care during these circumstances?**
**5.1** Is there anything you have tried as a practice (e.g. proactive calling of at-risk patients)—what has worked/not worked so well? Have there been any unintended consequences?
**5.2** Is there anything you would like to do to—what would be the next steps in making this happen and is there anything that would act as a barrier?
**5.3** What would be the role for learners (eg medical students, F2s) placed in primary care to assist with the management and safety of complex elderly patients?
**6 Lastly, is there anything that is stressful or worries you about the primary care response to COVID-19 or your role within it?**

### Recruitment and study procedures

Participants were recruited using a purposeful sampling technique to ensure their in-depth understanding and familiarity with changing policies [24]. All participants must have been actively involved in direct patient care as GPs in NWL before and during the COVID-19 pandemic. Email invitations were distributed in two rounds, separated by 12 days during the period from 6 to 22 May 2020, amongst 294 GPs working across 133 practices in seven Clinical Commissioning Groups (CCGs) in NWL.

Fourteen interviews were sufficient to reach data saturation; no new information or themes-related to the study objectives were evolving from extra interviews; and the state of “informational redundancy”, as described by Sandelowski [25], was achieved [26]. Eight interviews were conducted after the first round of email invitations, followed by six more interviews after the second round.

### Patient and public involvement

It was not feasible to directly involve the public in this study due to the challenging circumstances of COVID-19. We investigated the GPs’ perspectives on the impact of COVID-19 policies on patients, while we would encourage other research to explore the subject from patients’ perspectives, where public involvement should be prioritised.

### Data collection

Individual interviews were conducted virtually between 7 and 22 May 2020 using online platforms, due to the social distancing regulations in the UK during that period. The interviews lasted approximately 30 minutes and were video recorded. The researcher (AA) who conducted all the interviews had a clinical background as well as previous qualitative research experience in similar settings.

All participants were informed of the study confidentiality and anonymity. They were asked to reflect on the general impact of the COVID-19 policies in primary care practice, and then probed on the management and safety of elderly complex patients from different aspects. At the end of the interviews, the GPs had the chance to give suggestions for improvement and to reflect on their worries and stressors.

This study was assessed by Imperial College Research Ethics Committee (ICREC) to be a service evaluation, which does not require an NHS Research Ethics Committee approval [27]. All participants received a detailed study information leaflet with the invitation email, they had the opportunity to ask about any relevant clarifications prior to the interview, and they all provided informed verbal consent.

### Data analysis

The interviews were transcribed and manually analysed in an anonymous form using NVivo software. A directed content analysis approach was adopted to analyse the interview transcripts [28]. Two researchers with relevant experience (AA and PA) critically discussed the data to generate a preliminary coding scheme. The codes were furtherly appraised by the other two researchers (SK and RP) for refinement and to ensure its practicality based on their extended clinical experience in primary care.

The final coding scheme was approved by the four researchers and generated new major themes on a taxonomy of consequences from the primary care COVID-19 policies. The results will be presented following these new themes, which state the GPs’ perspectives, supported by quotes from the participants.

### Data validation

Ensuring validity has been considered from the interview guide development stage, through to data handling and analysis, to the interpretation and presentation of results. Two relevant and valid theoretical frameworks were integrated to develop the interview guide. The interview processes were pilot-tested with three experts in the field before commencing the main interviews.

Data analysis was a continuous process along with the study procedures. The outcomes from each interview were used to enrich the discussion in the following ones, without changing the interview’s core structure and design, until data saturation was reached. The analysis was conducted using NVivo software to ensure accuracy. The four authors have discussed and agreed on the final coding scheme and themes at different stages as explained in the data analysis section. The full code book of the analysis is provided as an appendix (S1 Table).

## Results

Fourteen interviews were conducted with practising GPs in NWL locality. Participants’ average experience was 14 years working for the NHS, which made them familiar with current systems and capable of identifying gaps.

“*The change has been huge*, *and we may never go back to the pre-COVID NHS*, *or primary care like before ever again*.”

(GP-9)

### Major themes and key outcomes

**Fig 1:**
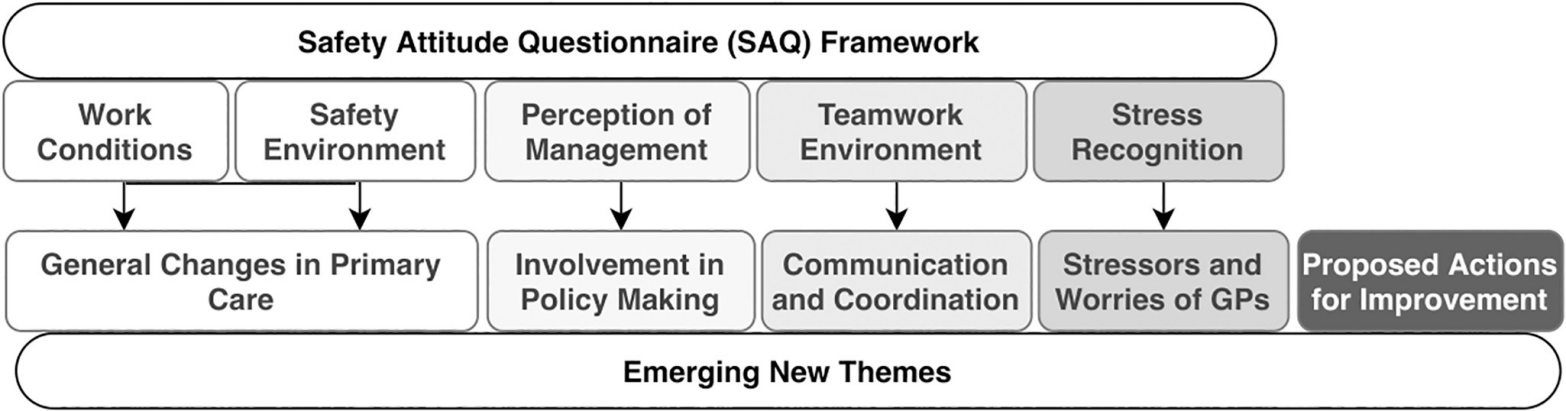
Illustration of the link between the SAQ framework and the study’s emergent themes.

Five major themes were identified from the data analysis that highlight GPs perceptions of how COVID-19 policies have impacted the safety and management of complex elderly patients (Fig 1). Four themes have been mapped onto the SAQ framework [22] to help interpret the data, and an additional fifth theme has been introduced to present the GPs’ insights on how to improve the existing primary care model from a frontline workers’ perspective.

GPs described the consequences of COVID-19 in primary care as overlapping and interconnecting over time. The necessity of adopting a multidisciplinary approach to overcome these challenges was a consensus amongst the participants.

“*…we need to stop thinking about targets*, *blood pressure targets*, *diabetes targets*, *and so on; I think we need to tailor the care to the patients*, *and their needs and wishes*.”

(GP-13)

### General changes in primary care

The interview data suggests how COVID-19 policies have impacted primary care at three levels, patients’ behaviour, healthcare professionals, and the overall work conditions; with an interplay between these, which ultimately impacts the quality and safety of service delivery.

GPs reported how patients’ behaviour has changed, with patients more willing to practice self-management for minor conditions. Although this may be seen in a positive light, it is thought this change in health seeking behaviour was driven by the patients’ fear of contracting COVID-19 if they attend healthcare facilities, rather than an increased sense of empowerment and activation.

“*A lot of patients are afraid… they are very afraid of going to hospitals in case they catch COVID-19*”

(GP-13)

GPs described how COVID-19 policies directed their focus towards acute illnesses, such as acute infections, flu-like symptoms, and the likely-manifestations of COVID-19 infection, while monitoring chronic conditions was seen as less of priority. However, primary care teams have been proactive in addressing this gap and have themselves developed approaches to continue chronic disease monitoring.

“*…and for the first time ever we are proactively ringing those over 70s*, *we are chasing them saying; are you OK*?”

(GP-5)

The work conditions in primary care were also felt by GPs to have significantly changed during the COVID-19 pandemic. Online consultations have become the norm, while the number of face-to-face appointments has considerably reduced. GPs did not seem to mind the change, as they acknowledged the need to reduce the risk of infection, and they also reported a sense of acceptance from patients. GPs described how appointments for remote consultations became more available and easier to book; however, they highlighted the challenges of conducting effective consultations remotely.

“*I think that we are always going to miss something by not being in the same room with patients*.”

(GP-5)

The GPs described a need for more resources, Personal Protective Equipment (PPE) and communication appliances, such as desktops and cameras, to maintain best practice. The participants expressed their perception that primary care was relatively neglected by statutory authorities and its role in the pandemic response was undervalued.

“*I think primary care has been a little bit forgotten*, *especially when it comes to the personal protection equipment*.”

(GP-8)

#### Impact on complex elderly patients

GPs echoed the legitimate attention that has been placed on the management of COVID-19 patients and how the new policies have affected the care of all patient groups. Concurrently, they emphasised some concerns of how the changes in policies have particularly impacted the safety of complex elderly patients.

“*I think every complex elderly patient is different and unique…*. *There is not really a one-answer fits all for elderly people*.”

(GP-12)

The fear of contracting COVID-19 in healthcare facilities discouraged elderly patients from communicating with their doctors, even remotely, to avoid being called in to the surgery or hospital. GPs described such issues and the lack of face-to-face consultations as potential risks of misdiagnosis, delayed care, missing early signs of disease deterioration, and the inability of monitoring and updating medications for patients with complex conditions.

“*A lot of unnecessary clinical risks because we (GPs) were not able to get investigations done for patients*, *or to see them as we normally would*.”

(GP-7)

“*There are lots of more routine things which we keep having to postpone like blood tests for medication monitoring and more routine investigations that seem just to be put off and off and off*.”

(GP-12)

The participants commented that elderly people are more likely to have multimorbid diseases, which requires an integrated approach with continuous follow-up to ensure their safety. They stated that remote consultations make it more challenging to monitor and manage those patients. GPs emphasised the value of family, social care, and community in supporting complex elderly patients to help with their mental wellbeing and their ability to engage with technology.

“*So many people were willing to help with sort of food pickups*, *delivering medications*, *and other stuff*, *and that has made me realise*, *actually*, *that this nation is willing to look after the elderly*.”

(GP-1)

There was also concern over the accessibility of remote consultations, especially amongst elderly patients and the potential to misinterpret public health messages around the need to stay at home meaning avoiding medical attention. GPs deployed extra efforts in order to proactively reach out to complex elderly patients. As the participants described, some patients even thought that healthcare services were totally suspended for all non-COVID conditions at the beginning of the pandemic, which was not the case.

“*They (patients) might be waiting on things for a while*, *like blood in stool or symptoms of cancer*. *Previously*, *it was easy to come to us*, *while now*, *they do not come as much*. *This is a real issue*.”

(GP-9)

### Involvement in policy making

The urgent nature of the pandemic and the need to have prompt reactionary policies in-place meant GPs had reported a limited level of involvement in policy making. Nevertheless, they stated that the UK policies seemed to be reflective and responsive to the reality, despite some initial delays.

“*Sometimes we might have already put stuff in place*, *and then it comes down as a requirement later on*.”

(GP-10)

The participants commented that the policies did not specifically address the needs of complex elderly patients, except for shielding recommendations for a relatively small proportion of highly vulnerable patients. In practice, GPs prioritised some patient groups who needed more attention such as, those living alone; those who need regular monitoring for their medications; multimorbid patients; elderly in care homes; patients with chronic medical conditions; patients with dementia; and the shielded groups.

### Communication and coordination

GPs reported some difficulties in the use of technology in setting up a new effective triage system, and in engaging patients in using technology in these new systems. They stated that both sides need to familiarise themselves with the “new normal”. The need for a continuous Information Technology (IT) support was highlighted as essential for maintaining this service. Face-to-face consultations were suggested to be an essential option for particularly vulnerable patient groups.

“*Obviously*, *we have started with a new technology now…You worry that your IT skills are not good enough to do all these new technologies*.”

(GP-3)

Within each practice team and across CCGs, the communication was reported to have improved over time. Participants described how the online coordination saved time and boosted the ability to keep healthcare teams connecting. However, a delay and some lag in coordinating services with other teams, such as referrals to secondary and tertiary care, were also observed.

“*I think it has brought everyone together*, *and it’s much more effective because everyone can attend wherever you are*”

(GP-4)

“*Infodemic*” is an expression that was used by one of the participants to describe the GPs’ everyday pressure of receiving a vast amount of new information, recommendations, and updating policies. The GPs felt responsible to remotely circulate these updates within their teams to ensure best practice, in addition to coordinating services with other teams and authorities to deliver integrated care. There was inconsistency amongst participants whether this phenomenon was beneficial or led to more uncertainty and confusion.

“*It’s not just a pandemic that we’re going through*, *it is an “Infodemic” as well*.”

(GP-9)

### Stressors and worries of GPs

The vast majority of participants’ worries were towards their patients rather than themselves. They worried about the impact of the new COVID-19 policies on their ability to engage with patients early to prevent serious complications or deterioration. This has been due to either patients’ fears of engaging with healthcare, or a delayed response due to the online-first model of care.

“*I had a case of a lady who had a fall*, *decided not to get in touch with us*, *and ended up having headaches and finally became drowsy*, *and called 999*. *When she went to the hospital*, *she had raised INR and subdural haemorrhage*.”

(GP-2)

They were also stressed about the future of primary care and what the “new normal” will look like. Other concerns regarding this shift to digital consultations included: inequality, safety, and the quality of care provided to elderly patients in different communities from different socioeconomic classes.

“*I am worried that we may revert and maintain this model of pure virtual and to lose the face-to-face contact that I really value as a clinician with my patients*.”

(GP-10)

### Proposed actions for improvement

Participants highlighted the need to consider the long-term impact of current policies and offered suggestions to overcome noticeable gaps in primary care. Prioritising the suggestions was not feasible as participants emphasised the necessity of considering different improvement actions to run in parallel to improve the service.

“*I think there has been an appetite to use this window of opportunity to make some permanent changes… The old model of primary care was quite outdated*, *and it didn’t really suit the actual needs of most people*”

(GP-14)

Table 2 summarises the participants’ suggestions, categorised based on the four adapted themes from the SAQ framework [22] and supported by quotes from the interviews. Many of these suggestions are in-place local initiatives applied in a narrow scale amongst a specific GP practice, whereas GPs advocated for the dissemination of these initiatives in a broader scale after observing their local success. Other suggestions were presented by participants as new proposals for improvement based on their experiences.

**Table 2:** Suggested improvement actions to overcome the identified challenges and gaps.

Categories	Actions for improvement
**General changes in primary care**	• Ensure that each elderly patient has a supportive relative, carer, or advocate to help him/her use technology and conduct video consultations where possible. If not possible, face to face consultations should be accessible.
	• Establish awareness campaigns for patients on how to access services
	• Explore possibility of disseminating specialised GP practices to deal exclusively with COVID patients, which would eliminate the infection risk for non-COVID patients in other practices
	• Incorporate remote health services within individual patient care plans and guideline documents
	• Consideration of how locum GPs can be better supported, and their services incorporated into the health workforce during a pandemic
	• Ensure elderly patients are allocated with a regular healthcare professional for continuity of care
	• Establish a proactive “Rapid Frailty Service” or “Frailty Teams” particularly serving complex elderly patients at homes where this is not already operating.
	• Involve medical students to support GPs in primary care
	• Establish “Elderly Clinics” to provide dedicated time to focus on the needs of complex elderly patients with longer appointments
	• Adopt an organised proactive approach in follow-up of complex elderly patients in primary care
**Involvement in policy making**	• Establish a one-voice strategy to deliver updates and new policies for doctors
	• Define a clear pathway to transfer instructions and control the information flow amongst health care professionals
**Communication and coordination**	• Boost the capacity of community nursing to support elderly patients at home
	• Establish robust coordination strategy with social care services
	• Employ healthcare assistants to coordinate different services for complex elderly patients at home
	• Create multidisciplinary elderly care teams to have regular meetings with each practice for high risk complex patients
**Stressors and worries of GPs**	• Ensure that GPs and health professionals in primary care are not overloaded with administrative duties in order to dedicate their time to their patients
	• Involve health professionals and patients in decision making around policies to ensure their needs and concerns are addressed

For instance, participants proposed a change in the dissemination strategy of information and guidelines.

“*Instead of this sort of scatter gun approach of emails which eventually people (GPs) ignore*, *they needed to be perhaps a one-voice… it needs to be a clearer root of how information disseminates*.”

(GP-5).

Another example, GPs emphasised that the coordination of services between primary care and social care is key to provide safe patient-centred care to elderly people during the current COVID-19 pandemic and beyond.

“*We have got better relationships through this with our district nurses and social care teams*, *so they are calling us much more*.”

(GP-5).

In terms of implementation, barriers included the need to share responsibility between healthcare professionals, patients, and decision makers to overcome the challenges. Participants suggested that healthcare professionals should focus on becoming used to “the new normal” while maintaining best practice. From the GPs’ perspective, policy makers need to dedicate extra resources and efforts to make their decisions reflective of changes on the ground.

“*Sometimes what we are trying to do*, *especially with entrepreneurialism and innovation*, *needs to be tested in the actual environment if it is doable*. *Testing the solutions in old fashion ways*, *such as extended clinical trials*, *may take years*, *while the problems are now*.”

(GP-8)

## Discussion

### Summary

This study has described GPs’ perceptions of how COVID-19 policies have impacted primary care. In the UK, a total remote triage system and remote consulting were rapidly adopted; something which has been sought by the NHS for many years [11, 29]. GPs commented how some of the “unnecessary workload” on GPs was reduced, however, GPs were expected to adapt to new systems at pace. Although the online communication amongst practice teams and across CCGs was felt to be more efficient, participants felt inhibited by the daily information overflow. They called for a one-voice strategy to deliver policy updates.

Participants reported how online appointments became more accessible to patients, despite the challenge of using technology. GPs described the benefit of family and community support for elderly patients to effectively engage with digital primary care, however, ensuring there is flexibility to provide alternative routes for those unable to access or use technology, to ensure they are not disadvantaged. More strict safety measures were reported to be in place when inviting an elderly patient to a face-to-face appointment, referring to specialist care in hospitals, or conducting a home visit to protect at-risk patients from contracting COVID-19. It was felt that some patients avoid seeking medical advice due to the fear of contracting COVID-19 while receiving healthcare services, which led to serious complications in some cases.

Participants felt that there have been efforts made to make policies responsive to needs, however, healthcare professionals asked for more involvement in decision making. A key message from the study was the need to emphasise and ensure appropriate support for the central role that primary care plays in maintaining the safety of complex elderly patients. As the COVID-19 policies had a multifactorial impact on healthcare, a multidisciplinary approach that involves healthcare, social care, and community support was felt to be needed like never before to fill the gaps.

**Fig 2:**
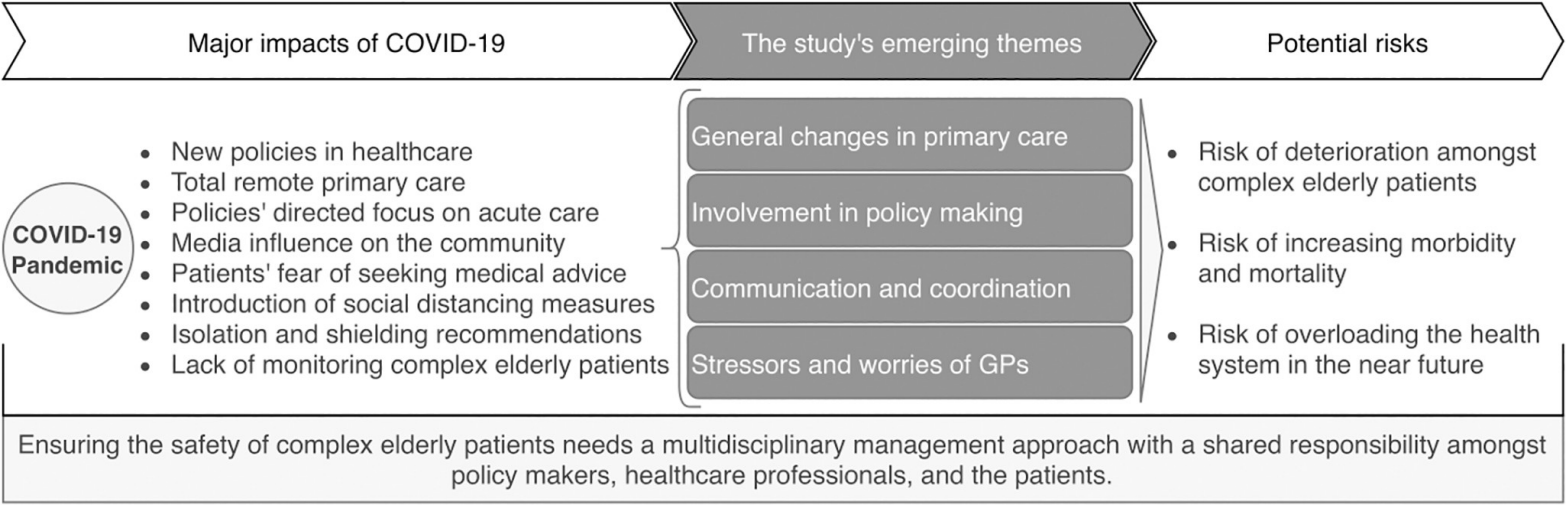
The flow of COVID-19 pandemic’s consequences in the UK, from GPs’ perspective, and how these are linked to the study’s emerging themes.

Fig 2 illustrates the link between the study’s four adapted themes from the SAQ framework [22], and the reported challenges and potential risks of the COVID-19 pandemic in the UK from the GPs’ perspective. We asked the participants in the interviews to propose avenues for tackling the challenges, which are concluded in Table 2 under the additional fifth theme. These avenues have been in the form of suggestions or existing actions that were experienced by GPs.

### Comparison with existing literature

The study shows that there are multiple factors that GPs perceive as influencing the safety of elderly complex patients in primary care along with the COVID-19 policies. These factors extended to community and social aspects, which have been shown to impact healthcare [30].

Interviews described how the changes-related to the elderly people, in terms of shielding and self-isolation, have impacted the patients’ access to healthcare services. The lack of social communication and community networking was thought to have a dramatic effect on patients’ mental health as well as engagement with their own health needs and management [31].

The wider health determinants such as socioeconomic circumstances and policy changes have been significantly affected by the COVID-19 pandemic [32, 33]. GPs discussed how those determinants changed their daily work environment, the way they manage their patients, and their perception of the future of primary care in the UK. Healthcare professionals and policy makers should realise how to adapt with these changing determinants to provide best patient-centred care in all contexts [33].

### Strengths and limitations

This study integrated diverse perspectives of practising GPs who have been delivering primary care in the NHS, before and during the COVID-19 pandemic. This significant experience made the participating GPs familiar with the traditional NHS system and aware of the baseline state of health within the community.

Many participants were also involved in managerial and research work, beside their clinical practice, which enriched their experience and deepened their ideas on system gaps and proposing practical solutions. However, there is still a need to investigate the perspective of GPs who are exclusively practising clinically.

The study timing, after 2 months of introducing the UK COVID-19 Action Plan published on 3 March 2020 [34], allowed the participants to adapt to the new changes and reflect on the impact on their patients’ care and health. This enhanced the value of suggested improvements, which considered service integration and sustainability in the post-COVID era.

All participants were GPs working in the NWL locality, which may limit the generalisability of outcomes. London was initially worse hit than other UK regions and has a very diverse population and was perhaps subject to more stress during the peak of the pandemic. However, given the implementation of the COVID-19 policies nationally, these findings may still be applicable going forwards and useful for other areas in the UK. Additionally, developing a framework-guided design for the study and the thematic analysis favours the study’s replicability in similar contexts.

This study presents only the GPs’ perspective; however, other research is needed to explore the subject from other healthcare professionals’ viewpoints, such as community nurses and healthcare assistants. There is also a vital need to work in partnership with our patients to explore their perspectives and experiences of health services during the pandemic. The challenging time of conducting this study limited the possibility of expanding the interviews to include other professionals, community members and patients, which should be feasible later as an avenue for research.

### Implications for research and practice

In mid-June 2020, the WHO European Regional Centre for Primary Care highlighted the need to reassess essential primary care services provided for non-COVID patients during the pandemic [35]. It is crucial to establish a resilient system that is accessible, feasible, and efficient for everyone to obtain basic care, leaving no one behind [36].

Our data has highlighted the importance of integrating social care and community support in delivering patient centred primary care services during the crisis [37]. This could help free up the resources and time to enable those patients who do need specialist or face-to-face healthcare to obtain timely services [38, 39].

GPs highlighted the need of dedicating extra resources and tools to maintain effective communication channels with their patients and medical colleagues. These tools need to be accessible and user friendly for all patients to avoid any inequalities in using technology or marginalisation of underprivileged communities [40, 41].

The participants also advocated for assessing and disseminating the on-going initiatives in the UK that adopted multidisciplinary approaches and were deemed useful during the pandemic. One example is the NHS Rapid Response Teams that deliver urgent care at home for those in need [42]. This can help overcome some of the limitations of remote consultations, particularly amongst complex elderly patients, allowing, when required, for patients to be examined or closely observed.

Given that fact this study presents the perspective of GPs working in NWL, more studies should be considered to investigate the perspectives of other healthcare professionals across the UK. There is still a need for more innovative solutions with shared responsibility across different dimensions of care to enhance the safety and quality of service delivery during the pandemic and beyond [43]. These implications for research and practice are particularly relevant given the potentially prolonged period of measures to combat COVID-19.

### Conclusion

The COVID-19 policies have affected all levels of healthcare in the UK, particularly primary care as a major system entry point. As reported by GPs, the dramatic reshaping of primary care from a face-to-face to an online-first model has provided opportunities to make primary care more available and coordinated during the crisis. However, an overreliance on using technology poses a potential risk of inequality concerning accessibility. It may particularly affect elderly patients and those who may struggle in using the newer communication tools to carry out medical consultations. This risk could be mitigated by encouraging the integration between health and social care and advocating for family and community support, especially for elderly patients. The abrupt transformation in the care model has also introduced potential risks in terms of information management and good clinical practice. Patients should be encouraged to seek medical advice, when needed, and be assured that appropriate mitigation strategies to reduce the risk of contracting COVID19 have been applied. Although this new model of primary care delivery in the UK may, to some extent, remain in place, it is vital to keep alternative traditional routes open for those unable to access or use technology.

## Supporting information

S1 Table The code book of data analysis conducted in Nvivo software. (DOCX)
