# Supplementary material for: Management and patient safety of complex elderly patients in primary care during the COVID-19 pandemic in the UK—Qualitative assessment
Source: PLoS One. 2021 Mar 29;16(3):e0248387. doi: 10.1371/journal.pone.0248387 (PMC8006979; doi:10.1371/journal.pone.0248387)
Supplement: S1 Table — (DOCX) [file pone.0248387.s001.docx]

*S1 Table: the code book of data analysis conducted in Nvivo software*

| **Name** | **Description** | **Files** | **References** |
| --- | --- | --- | --- |
| **1. GENERAL CHANGES IN PC** |  | **14** | **87** |
| Impact on health professionals |  | 5 | 14 |
| Changes in care plans and clinical decisions |  | 3 | 5 |
| Staff shortage & Preparedness issues |  | 4 | 9 |
| Patients' behaviour |  | 10 | 16 |
| Acceptance of care | All patients | 8 | 8 |
| More self-management | All patients | 6 | 8 |
| Work conditions |  | 14 | 57 |
| Changes in practice generally | General changes felt in the GP duties | 11 | 21 |
| Notable changes in daily work |  | 8 | 18 |
| Positive & Negative changes |  | 12 | 18 |
| Negative working conditions |  | 7 | 8 |
| Positive working conditions |  | 7 | 10 |
| **2. IMPACT ON COMPLEX ELDERLY PATIENTS** |  | **14** | **206** |
| Changes in care for the target group |  | 14 | 94 |
| Acceptance of care |  | 9 | 14 |
| Avoiding to contact the clinic |  | 10 | 18 |
| Care homes |  | 4 | 8 |
| Challenges from elderly side |  | 1 | 1 |
| Community support |  | 7 | 12 |
| Distinctions compared to services for other groups |  | 8 | 14 |
| Getting more care |  | 4 | 6 |
| Inequality in care |  | 1 | 1 |
| Urgent medical attention request - response |  | 4 | 5 |
| Using technology |  | 9 | 10 |
| Clinical management-related issues |  | 12 | 40 |
| Care strategies |  | 5 | 5 |
| Delayed presentation |  | 10 | 17 |
| Delayed response and care |  | 4 | 5 |
| Diagnosis issues |  | 2 | 2 |
| Follow-up actions |  | 4 | 4 |
| Referral issues |  | 3 | 3 |
| Uncertainty in clinical decision and management |  | 1 | 2 |
| Home visits |  | 8 | 11 |
| Impact on elderly patients |  | 13 | 37 |
| Isolation impact on patients |  | 8 | 10 |
| Negative impact on elderly patients |  | 5 | 8 |
| Positive impact on elderly patients |  | 5 | 7 |
| Shielding impact on patients |  | 7 | 12 |
| Safety environment |  | 9 | 24 |
| PPE shortage and usage |  | 2 | 4 |
| Safety at surgeries |  | 4 | 7 |
| Safety in home visits |  | 4 | 4 |
| **3. COMMUNICATION & COORDINATION** |  | **14** | **157** |
| Limited face-to-face consultation | All patients | 9 | 17 |
| Negative points |  | 9 | 20 |
| Online consultations |  | 12 | 41 |
| difficulties in using technology from doctors |  | 2 | 2 |
| negative impact |  | 9 | 16 |
| phone consultation |  | 5 | 5 |
| positive impact |  | 8 | 10 |
| video consultations |  | 3 | 4 |
| Patient-GP communication |  | 9 | 14 |
| Positive points |  | 11 | 22 |
| Staff communication |  | 12 | 30 |
| Communication with the secondary and tertiary care |  | 6 | 6 |
| GP communication with CCG & PCN |  | 7 | 9 |
| GP communication with the clinical team |  | 10 | 15 |
| Teamwork environment |  | 8 | 13 |
| **4. DECISION MAKING** |  | **13** | **37** |
| Involvement in decision making |  | 13 | 29 |
| At the practice level |  | 2 | 2 |
| Complicated too long process |  | 2 | 2 |
| Considering elderly patients in policies |  | 7 | 8 |
| No involvement |  | 6 | 9 |
| Irrelevant work overload |  | 2 | 2 |
| Prioritised patient groups in policies |  | 5 | 5 |
| neglection for elderly people |  | 3 | 3 |
| Updated with the new guidelines and protocols |  | 1 | 1 |
| **5. SUGGESTIONS FOR IMPROVEMENT** |  | **14** | **150** |
| Capacity support |  | 3 | 4 |
| Effective communication tools |  | 7 | 11 |
| Funding |  | 2 | 3 |
| Implementation barriers |  | 12 | 20 |
| Initiatives already in-place |  | 10 | 11 |
| Involvement in decision making |  | 1 | 1 |
| Medical student volunteers |  | 14 | 46 |
| benefits to the students |  | 3 | 3 |
| challenges in practice |  | 4 | 5 |
| suggested roles |  | 12 | 18 |
| worries |  | 8 | 15 |
| Monitoring strategies |  | 4 | 5 |
| New approaches |  | 10 | 15 |
| Proactivity in care |  | 8 | 13 |
| Sub-groups of concern | Among complex elderly patients | 7 | 15 |
| care home residents |  | 2 | 3 |
| Shielded patients as a priority group |  | 5 | 5 |
| specific disease groups |  | 7 | 15 |
| specific medication |  | 3 | 3 |
| unaccompaied elderly people |  | 7 | 8 |
| Updating records |  | 2 | 2 |
| **6. STRESS RECOGNITION** |  | **13** | **54** |
| General stressors |  | 9 | 20 |
| Stress relieving strategies |  | 2 | 2 |
| Worries for the future practice |  | 7 | 16 |
| Worries related to patient care |  | 8 | 14 |
| **EXAMPLES** |  | **11** | **24** |
| Fear of seeking medical advice |  | 5 | 9 |
| Non-involvement in decision making |  | 1 | 1 |
| Poor referral system |  | 3 | 3 |
| Questionable decisions by authorities |  | 1 | 3 |
| Relaxed follow-up and monitoring |  | 3 | 3 |
| Strategies to overcome challenges |  | 3 | 3 |
